# Design and Performance of Laser-Pumped Cs-Magnetometers for the Planned UCN EDM Experiment at PSI

**DOI:** 10.6028/jres.110.021

**Published:** 2005-06-01

**Authors:** S. Groeger, G. Bison, A. Weis

**Affiliations:** Université de Fribourg, Chemin de Musée 3, 1700 Fribourg, Switzerland; Paul Scherrer Institute, 5232 Villingen PSI, Switzerland; Université de Fribourg, Chemin de Musée 3, 1700 Fribourg, Switzerland

**Keywords:** high precision magnetometry, laser spectroscopy, optical detected magnetic resonance, optical pumping

## Abstract

We designed laser-pumped cesium vapor magnetometers in the *M*_x_ configuration for the control and stabilization of magnetic field fluctuations and gradients in a new experiment searching for a permanent electric dipole moment of the neutron. The intrinsic sensitivity of the device was determined to be 30 fT in a measurement bandwidth of 1 Hz, limited by laser noise. In the shot noise limit the magnetometer can reach a sensitivity of 7 fT for 1 s integration time. Test measurements of the stability of a 2 µT magnetic field in a threefold magnetic shield have revealed fluctuations on the order of 200 fT to 300 fT with integration times in the range of 2 s to 100 s. Those fluctuations were traced back to the stability of the power supply used to generate the magnetic field. The laser-pumped magnetometer fulfills the requirements set by the planned neutron electric dipole moment experiment.

## 1. Introduction

The precise measurement and control of magnetic fields and magnetic field fluctuations is an important step for experiments searching for a permanent electric dipole moment (EDM) of the neutron, and is one of the main factors limiting the accuracy. In a project approved by Paul Scherrer Institute (PSI) in Switzerland, a neutron EDM spectrometer is proposed [1] in which the neutron spin flip transition frequency is measured in four UCN storage chambers exposed to a homogenous 2 µT magnetic field. In addition, each neutron chamber has two compartments in which the neutrons are exposed to a static electric field of 15 kV/cm oriented parallel/antiparallel to the magnetic field. The signature of a finite EDM is a change of the neutron Larmor frequency synchronous with the rever sal of the relative orientations of the magnetic and electric fields. This experiment imposes very stringent constraints on the homogeneity and stability of the magnetic field.

Fluctuations of the magnetic field will be monitored by a set of 16 optically-pumped alkali-vapor magnetometers (OPM), using the property that the oscillation frequency of an OPM is proportional to the modulus of the local magnetic field. The magnetometers considered for the first generation of the neutron EDM experiment are self-oscillating Cs vapor magnetometers (OPM) in the Mx configuration [2,3], optically pumped by spectral discharge lamps (LpOPM). However, it was previously shown that the replacement of the lamp by a resonant laser can lead to an appreciable gain in magnetometric sensitivity [3,4]. In that spirit we have designed and tested a laser-pumped OPM (LsOPM) with a geometry compatible with the neutron EDM experiment under construction. In case the laser version shows superior performance it might be considered as an alternative to the lamp-pumped magnetometers in the EDM experiment. Here we present the design and discuss the performance of Cs-LsOPMs operated in a phase-stabilized mode.

## 2. The Magnetometer Setup

**Fig. 1:**
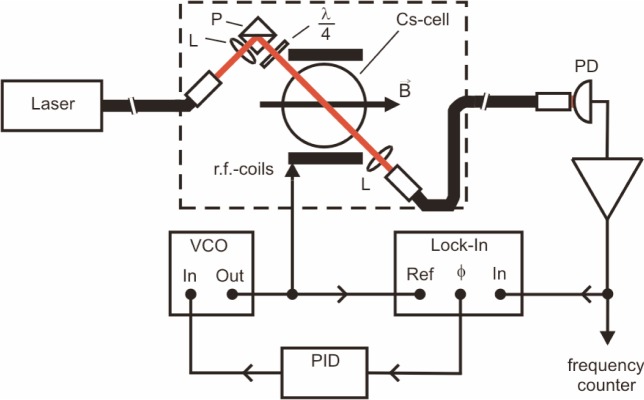
Schematic setup of the phase-stabilized magnetometer. The dashed box indicates the sensor head. L: lens, P: polarizing beamsplitter, *λ*/4: quarter-wave plate, PD: photodiode, VCO: voltage-controlled oscillator, PID: feedback amplifier. The stabilization system of the laser frequency is not shown.

The magnetometer consists of three parts (Fig. 1): a) a sensor head containing no metallic parts except the r.f. coils, b) a base station mounted in a portable 19″ rack drawer, which contains the frequency stabilized laser and all optics, and c) the frequency locking electronics. The sensor head is designed to fit into a tube of 104 mm diameter, coaxial with the 2 µT field, and has a total length of 242 mm. Its main component is a paraffin-coated, 7 cm diameter glass cell containing a droplet of cesium. The Cs atoms in the vapor are optically pumped by a circularly polarized light beam, oriented at 45° to the magnetic field. An extended-cavity diode laser, which is stabilized on the F = 4 → F = 3 hyperfine component of the D_1_ line at 894 nm wavelength by the dichroic atomic vapor laser lock (DAVLL) method [5], provides the resonant light which is guided from the base station to the sensor head via a 10 m long multimode fiber (800 µm core diameter). Note that a single diode laser can produce sufficient light power to drive all sensors in the experiment. The light transmitted through the cell is carried back to the detection unit by a similar fiber. The pumping process produces a polarization (magnetization) in the sample which undergoes a Larmor precession with the frequency *ω*_L_. The precession is resonantly driven by a weak radio-frequency (rf) field (of amplitude *B*_1_ and frequency *ω*_rf_) applied with a pair of circular coils surrounding the cell. The absorption of the pumping light depends on the direction of the magnetization with respect to the light direction. Therefore the resonant precession frequency appears as an amplitude modulation on the light power transmitted through the cesium vapor. The detection of the modulated transmission by a photodetector allows a direct and real time measurement of the magnetic field in terms of the Larmor frequency.

The system behaves like a classical oscillator: when an rf field is applied, the phase of the response, i.e., the AC component of the transmitted laser intensity depends on the detuning *δω*= *ω*_rf_ − *ω*_L_. For *δω* ≪ 0 the atoms follow the driving field adiabatically and thus the radio frequency and the light modulation oscillate in phase. For *δω* ≫ 0 the phase shift is −180°, while on resonance (*δω* = 0) it becomes −90°, while the modulation amplitude of the transmitted light reaches a maximum. In the phase-stabilized mode this dependency is used to lock the radio frequency to the Larmor frequency. The photodiode signal is analyzed by a dual-channel lock-in amplifier, referenced by the driving frequency of the rf coils produced by a voltage controlled oscillator (VCO). Either the phase or the dispersive in-phase output of the lock-in amplifier can be used in a feedback loop to lock the driving frequency to the Larmor frequency by stabilizing the phase shift to −90°. The temporal response of the phase-stabilized magnetometer to field changes, i.e., its bandwidth is determined by the response time of the feedback loop and was measured to be in the order of 1 kHz.

## 3. Performance of the Magnetometer

### 3.1 Intrinsic Magnetometric Sensitivity

**Fig. 2:**
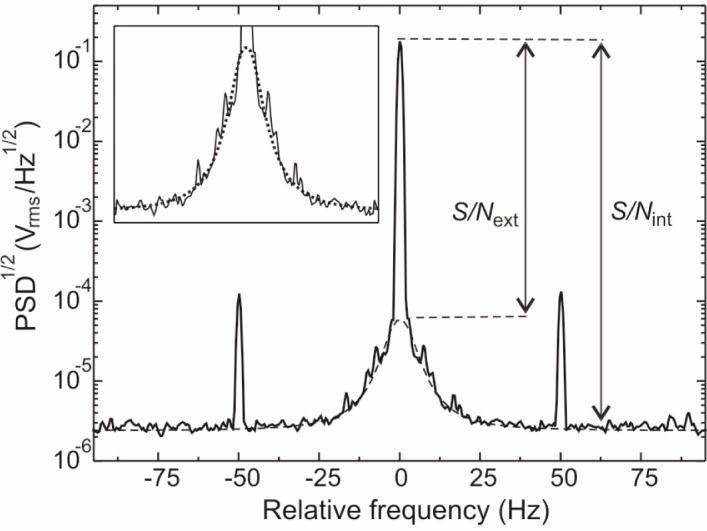
Square root of the power spectral density (PSD) of the magnetometer output frequency relative to the Larmor frequency of *ν*_L_ = 7032 Hz (averaged 20 times). The spectrum was measured with a 1 Hz resolution bandwidth. The signal-to-noise ratio *S/N*_int_ is approximately 66 000. The sidebands are due to imperfectly shielded magnetic field components oscillating at the 50 Hz power-line frequency. The dotted line is an approximation of the pedestal. The signal-to-noise ratio *S/N*_ext_ due to external field noise is found to be approximately 2600.

We define the intrinsic magnetometric sensitivity of the LsOPM in terms of the noise equivalent magnetic field (NEM). It is the square root of the power spectral density of magnetic field fluctuations at the magnetometer frequency. The field fluctuations produce a photo-detector signal equal to the power spectral density of the photocurrent at the same frequency, each integrated over a bandwidth *f*_bw_. The NEM *δB* is given by:

$$\delta B=\frac{1}{\gamma }\cdot \frac{\Delta \nu_{\text{HWHM}}}{S/N_{\mathrm{int}}},$$ (1)

where *γ* is a constant of approximately 3.5 Hz/nT for ^133^Cs. The half width of the resonance is ∆*ν*_HWHM_ and *S/N*_int_ the signal-to-noise ratio of the photocurrent modulation in the phase-stabilized mode. The signal *S* is the amplitude of the modulation. When measuring the intrinsic noise level, *N*_int_, care has to be taken to eliminate external contributions, such as drifts and noise from external magnetic field sources. We therefore infer the intrinsic noise *N*_int_ from a Fourier analysis of the photodiode signal when the magnetometer is operated under optimal conditions (Fig. 2). The central peak (the so-called carrier) is the oscillating magnetometer signal. It is superposed on a broad pedestal, which results mainly from a continuous distribution of sidebands due to imperfectly shielded low-frequency field fluctuations. The two discrete sidebands originate from interference of magnetic fields oscillating at the line frequency. The intrinsic noise level *N*_int_ is given by the noise floor in Fig. 2, integrated over the detection bandwidth *f*_bw_. It lies 50% above the shot noise level $\Delta I=\sqrt{2eI_{\text{pc}}f_{\text{bw}}}$ of the DC photocurrent *I*_pc_ in a bandwidth *f*_bw_ of 1Hz.

The optimum operating point was found for a laser intensity of 9 µW/mm^2^ and a *B*_1_ field amplitude of 2.7 nT, which yielded a linewidth *ν*_HWHM_ = 3.4(1) Hz and a signal-to-noise ratio *S/N*_int_ = 97 dB = 66 000. The corresponding intrinsic sensitivity is *δB*_int_ = 14.5 fT. If one assumes the noise to be white, the sensitivity scales with the square root of the bandwidth. Assuming the photodiode shot noise as ultimate sensitivity limit the LsOPM should reach a sensitivity of *δB*_SN_ = 10 fT in 1 Hz bandwidth, which corresponds to *δB*_SN_ = 7 fT for a 1 s integration time. The laser generated low frequency 1/*f* noise is mixed to the Larmor frequency and thus contributes to the noise spectrum. It was found to exceed the white noise level *N*_int_ by a factor 2 at the carrier frequency, which yields an intrinsic sensitivity of δB^*^_int_ = 29 fT. This contribution can be suppressed using an active stabilization of the laser power.

### 3.2 Application: Field Fluctuations in a Magnetic Shield

**Fig. 3:**
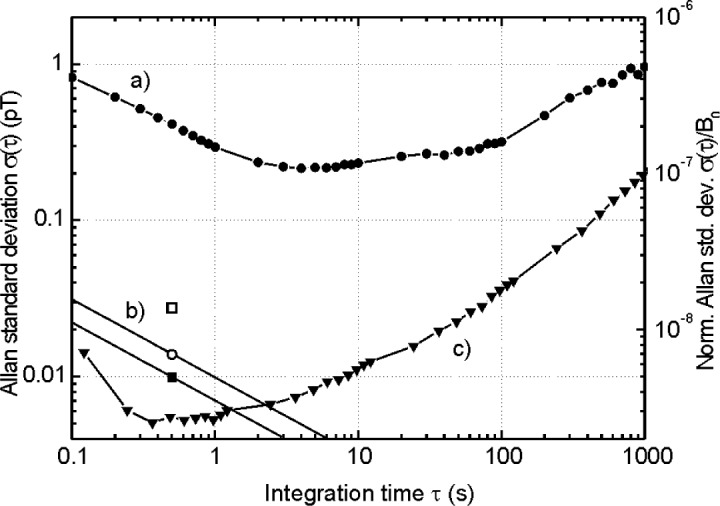
a) Allan standard deviation of the magnetic flux density inside the magnetic shield. b) Intrinsic sensitivity limit. δB^*^_int_ (square): sensitivity including 1/*f* noise of the laser power; *δB*_int_ (circle): sensitivity from experimentally determined white noise floor; *δB*_SN_ (filled square): anticipated sensitivity for shot noise limited operation. The slopes assume white noise. c) The triangles represent the light shift effect due to light power fluctuations. Solid lines in a) and c) are drawn to guide the eye. The dwell time of the frequency counter was 100 ms.

We used the LsOPM to measure residual field fluctuations inside a three-layer magnetic shield. The Larmor frequency was recorded as multiple time series of several hours with a sampling rate of 0.1 s. From each time series the Allan standard deviation [6] of the corresponding magnetic field inside the shield was calculated. A typical result is shown in Fig. 3. The observed fluctuations (curve a) are well above the intrinsic sensitivity level of the magnetometer (curves b). For integration times up to 1 s to 2 s the noise amplitude decreases as *τ*^1/2^, where *τ* is the integration time, indicating the presence of white field-amplitude noise. It can be characterized by a spectral amplitude of 413 fT/Hz^1/2^. With the estimated signal-to-noise ratio *S/N*_ext_ = 2600 (cf. Fig. 2) we calculate from Eq. (1) a field stability of 370 fT in a measurement bandwidth of 1 Hz, which is in good agreement with the value in the Allan standard deviation plot. The minimum field fluctuations were found to be slightly larger than 200 fT for an integration time of 4 s. The central region of the Allan standard deviation plot shows a plateau for integration times of 2 s to 100 s. It could be traced back to fluctuations of the 8 mA current producing the 2 µT bias field and corresponds to a relative stability of 10^−7^ for the power supply. The Allan standard deviations for integration times exceeding 100 s are due to slow drifts of laboratory fields which are not completely suppressed by the three-layer shield, which has a measured longitudinal shielding factor of 10^3^. The influence of light shift effects [7] due to light intensity fluctuations are measured in an additional experiment. Figure 3c shows that those effects are negligible at the present level of field stability.

## 4. Summary and Conclusion

We have described the design and performance of a phase-stabilized cesium vapor magnetometer with a measurement bandwidth of 1 Hz. The magnetometer has an intrinsic sensitivity of 29 fT, defined as the Allan standard deviation for a bandwidth of 1 Hz. This value can be reduced by a factor of 2 if the 1/*f* noise of the laser power can be lowered, e.g., by an active power stabilization. If the excess white noise can be reduced to the shot noise level a further increase of 1.5 can be obtained. Under these optimal conditions the LsOPM could reach a sensitivity of 7 fT for an integration time of 1 s. Field fluctuations of 200 fT to 300 fT were measured in a three-layer magnetic shield for integration times between 2 s and 100 s. Light shift fluctuations, against which no particular precautions, except for the active frequency stabilization of the laser, were taken, are one to two orders of magnitude smaller than the residual field fluctuations in the shield. The laser-pumped OPM described here will thus be a valuable tool for fundamental physics experiments and compares very favorably with state-of-the-art lamp-pumped magnetometers as it will be demonstrated elsewhere.
